# Supplementary material for: Seminoma and Embryonal Carcinoma Footprints Identified by Analysis of Integrated Genome-Wide Epigenetic and Expression Profiles of Germ Cell Cancer Cell Lines
Source: PLoS One. 2014 Jun 2;9(6):e98330. doi: 10.1371/journal.pone.0098330 (PMC4041891; doi:10.1371/journal.pone.0098330)
Supplement: File S1 — ZIP file containing DMRforPairs output for significant regions. Please start from the html files. (ZIP) [file pone.0098330.s008.zip › figures/19609.pdf]

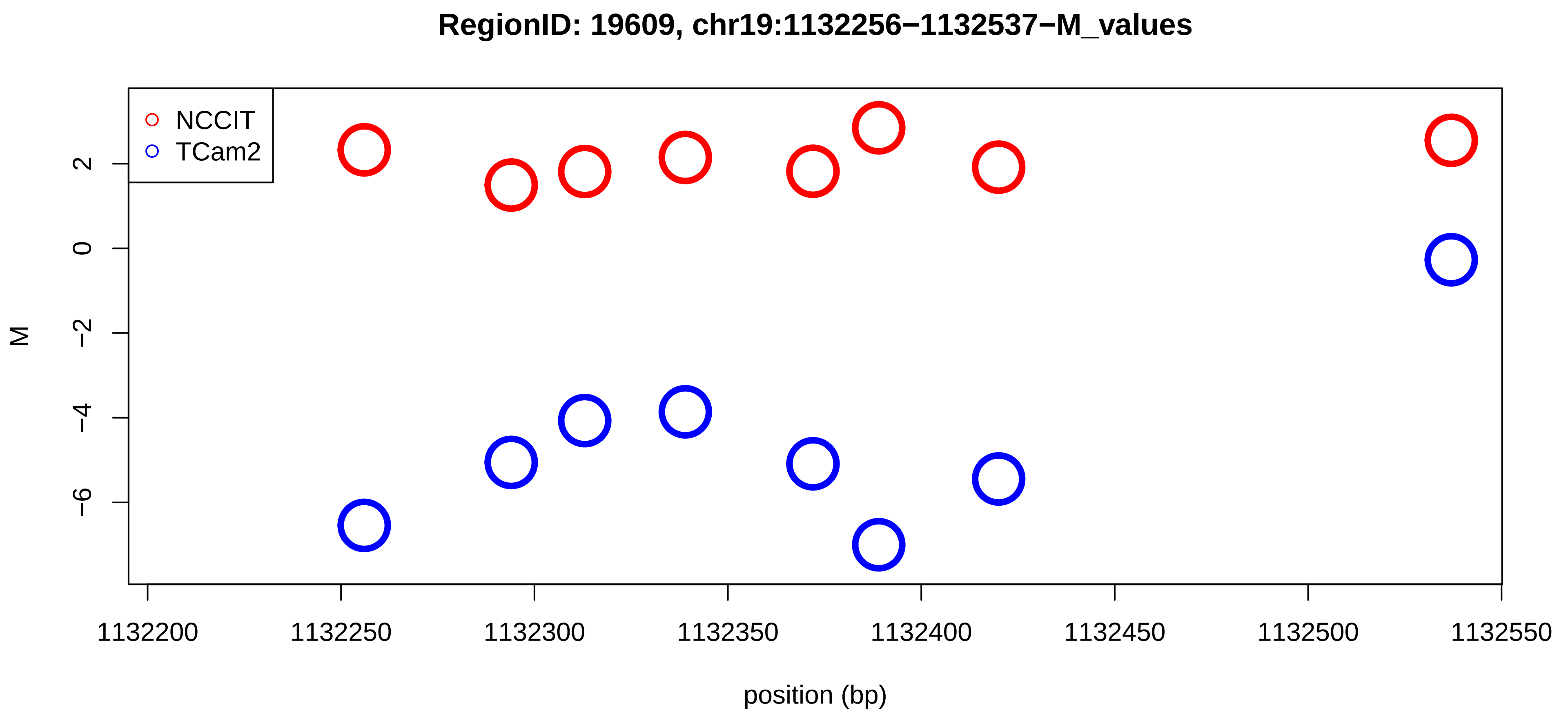
RegionID: 19609, chr19:1132256−1132537−M_values
NCCIT
TCam2
M
2
0
−2
−4
−6
1132200
1132250
1132300
1132350
1132400
1132450
1132500
1132550
position (bp)

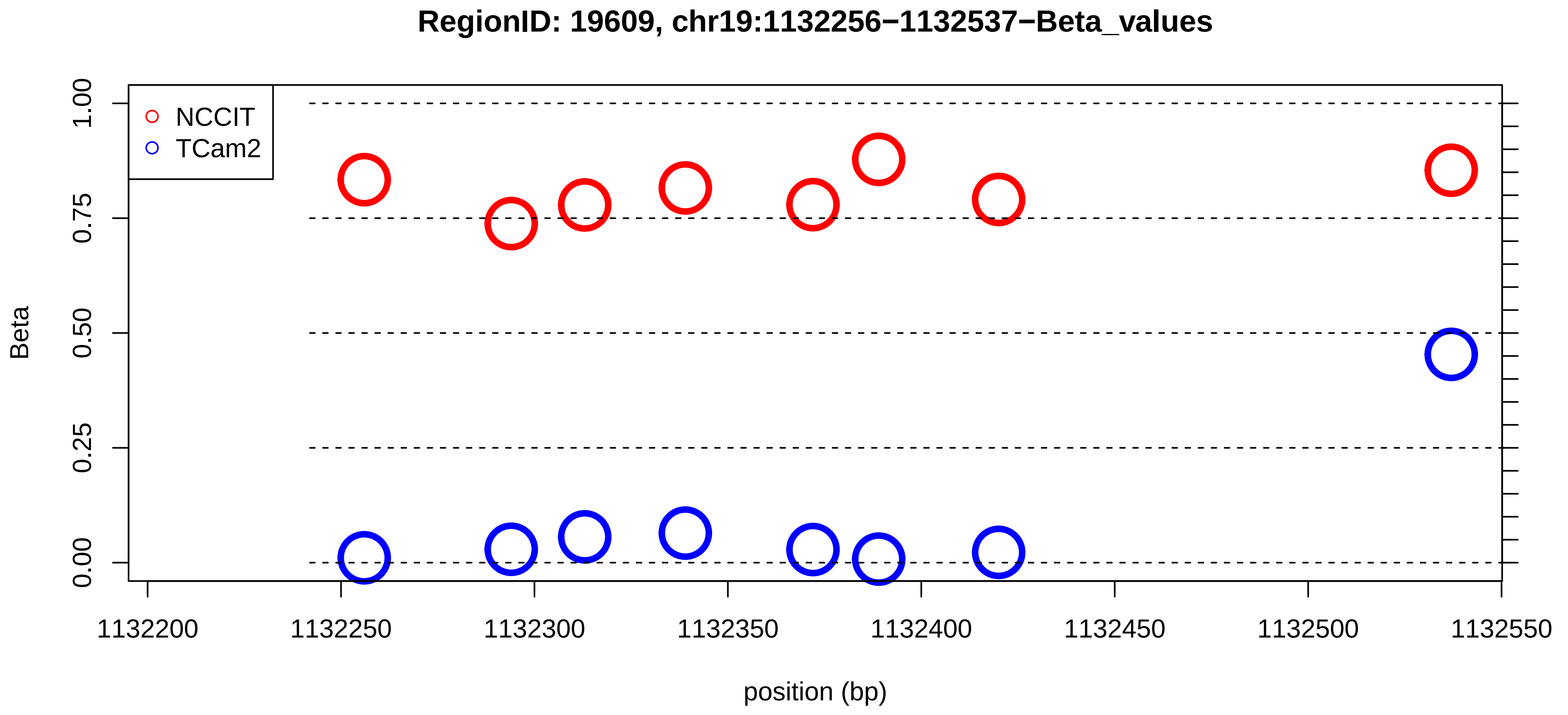
RegionID: 19609, chr19:1132256−1132537−Beta_values
NCCIT
TCam2
Beta
1.00
0.75
0.50
0.25
0.00
1132200
1132250
1132300
1132350
1132400
1132450
1132500
1132550
position (bp)
